# Can Dance and Music Make the Transition to a Sustainable Society More Feasible?

**DOI:** 10.3390/bs12010011

**Published:** 2022-01-10

**Authors:** Eva Bojner Horwitz, Kaja Korošec, Töres Theorell

**Affiliations:** 1 Department of Music, Pedagogy and Society, Royal College of Music, P.O. Box 277 11, SE-115 91 Stockholm, Sweden; kaja.korosec@ki.se (K.K.); tores.theorell@kmh.se (T.T.); 2 Center for Social Sustainability, Institution of Neurobiology, Care Sciences and Society, Karolinska Institute, SE-141 83 Stockholm, Sweden; 3 Department of Clinical Neuroscience, Karolinska Institute, SE-171 77 Stockholm, Sweden; 4 Stress Research Institute, Stockholm University, SE-106 91 Stockholm, Sweden

**Keywords:** dance, inner sustainability, mind-shift, music, transition

## Abstract

Transition to sustainability is a process that requires change on all levels of society from the physical to the psychological. This review takes an interdisciplinary view of the landscapes of research that contribute to the development of pro-social behaviors that align with sustainability goals, or what we call ‘inner sustainability’. Engaging in musical and dance activities can make people feel trust and connectedness, promote prosocial behavior within a group, and also reduce prejudices between groups. Sustained engagement in these art forms brings change in a matter of seconds (such as hormonal changes and associated stress relief), months (such as improved emotional wellbeing and learning outcomes), and decades (such as structural changes to the brains of musicians and dancers and superior skills in expressing and understanding emotion). In this review, we bridge the often-separate domains of the arts and sciences by presenting evidence that suggests music and dance promote self-awareness, learning, care for others and wellbeing at individual and group levels. In doing so, we argue that artistic practices have a key role to play in leading the transformations necessary for a sustainable society. We require a movement of action that provides dance and music within a constructive framework for stimulating social sustainability.

## 1. Introduction

In 2015, UN member states adopted the 2030 Agenda for Sustainable Development. They define ‘sustainable’ as “meeting the needs of the present without compromising the ability of future generations to meet their own needs” [1]. The 2030 Agenda encompasses 17 goals aimed at protecting the planet and achieving a higher quality of life [2,3]. These goals are aimed at pressing issues such as poverty, inequality, and climate emergency. To be able to achieve change quickly and efficiently, we argue that there is a need to understand how a state of ‘inner sustainability’ emerges. By ‘inner sustainability’, we mean people’s inner worlds: the values, beliefs, attitudes, identities and emotions that serve as the inner foundation for sustainable behaviors—behaviors that do not deplete people’s resources in the long run [4,5].

While the transition towards sustainability encompasses many changes to physical form and structure (e.g., energy supply, construction and transportation), we must not forget that it is also a psycho-social process [6]. A good deal of imagination and empathy is required to be able to understand how different unsustainable practices affect other people, or how they might affect them in the future. The development of such inner sustainability is likely to involve a variety of cognitive processes, including acute self-awareness and compassion, but also our social context and practices as groups: how do we need to listen to, communicate and collaborate with one another, so that we create a sustainable social environment? While these shifts “go deep” and may therefore involve significant time and attention, they are needed to accomplish more lasting changes than are possible through technical innovations and behavioral interventions alone [7] (for more detail on the relationship between different types of sustainability, see [7,8,9]). The inner transformation towards sustainability requires us to confront complexity and uncertainty, and thus taps into our capacity to deal with strong emotions, learn and adapt. As any change, it is closely related to individual and societal resilience, or the ability to utilize strengths and resources such as compassion towards others and as well as oneself [10]. The shift in mindset required to accomplish inner sustainability may rely in particular on pro-social values, emotions and skills, such as empathy, compassion and caring behaviors [11].

We have chosen music and dance due to their potency in interventions, the relationships between the two and their accessibility. Music and dance may offer a route to inner transformation though targeting compassion as a set of action-driven embodied processes [12]. Combinations of several such skills at the same time add further to emotional skill [13]. Thus far, we know that transformational states are triggered by combinations of music and dance movements [14]. The arts have been employed in interventions that transform the individual and social groups and Gabrielsson’s research provides examples of narratives in which inner transformational actions have taken place [15]. The transformative potential of the arts is likely based in their recruitment of the whole body (and senses) in rhythmic activity and play. According to Lauzon [16], music and dance are a form of time-ordered “trans-verbal play” in the domains of sound and movement, respectively. Many scholars maintain that there is overlap between definitions of music and dance: music may contain movement, dance may contain sound (see also Bojner Horwitz et al. [17]). The concept of Musikhé comprised dance, music and poetry in the forms of arts that were associated with some of the Greek Muses—Terpsichore (song and dance) and Erato (lyrics and love poetry)—which are typical examples of muses with double functions [14]. In some contemporary cultures, there is little conceptual differentiation between dance, music and movement. It is widely accepted that we are emotionally touched by both dance and music [14,18,19]. Researchers have argued that it is the feeling of rhythm in a dance movement that is the basis for the experience of music (“music is movement”). In this way, rhythm serves as the linkage between dance and music. Rhythm has a central role in the organization of behavior both within and between individuals in a group more widely [20].

The arts are necessarily embodied. We use ‘embodied’ in this article to refer to the embeddedness of the mind in the body and the body in its environment [21]. The Swiss musician and composer Emile Jaques-Dalcroze [22] describes the body as the superlative musical instrument: he believed all music originates in bodily signals and that training in rhythm is a method for connecting the body with music, forming an important part of all musical teaching. The teaching of musicianship often involves accompanying training of bodily awareness with breathing exercises, relaxation, and contemplation. Such practices are also fundamental to dance as an art modality [22].

Coordination of visual, auditive and motor stimuli occur in the brain during dance movements. Dance training links feelings with physical movements and this means that the dance movements become resonant with the feelings. Such coordination may arise in mirror neurons [23] (for a more detailed view of synchronization and coordination, see [24,25]). Musical practice sometimes appears to be more specifically focused on some bodily functions, such as finger movements. However, in musicians, more general brain structures that associate sound interpretation with emotions also seem to grow with practice on their instruments [26]. The scientific literature contains a greater number of articles that investigate the brain plasticity of musicians’ brains than the brains of dancers, likely due to the difficulty of combining neuroscientific measurement with whole-body movement [27]. A parallel observation has also been made regarding the balance of studies of music and dance therapy [28].

A further link between music and dance, and a possible clue as to why it is sometimes difficult to draw a clear line between them, is the amygdala. The amygdala is a part of the brain that regulates acute feelings, including those of depression, anxiety and stress [29]. Activity in the amygdala, which can be recorded by means of functional magnetic resonance imaging, increases in emotionally charged situations regardless of which modality (visual, auditive, tactile, etc.) the feeling arises in. This may explain why it is difficult to separate the modalities of music and dance from one another. Psychophysiological responses such as changes in blood pressure, heart rate variability and skin conductance are involved regardless of modality. In micro-phenomenological studies, a dance movement may be closely linked to inner sound impulses that are intertwined in our consciousness [17,30].

During prolonged music and/or dance practices, Giacosa et al. [23] found that different stimuli could influence nerve activities in different ways. During dance practices, the whole body’s nervous system is activated with the coordination of visual and auditive sensorimotor signaling, as well as with the AON (Action Observational Network) [31]. Long-term music practice, on the other hand, includes more specific control of auditive-motoric integration [32]. The authors observed this difference in nerve activation to be reflected in the growth of white matter (the links between the nerve cells themselves). Dance practice—compared to music practice—seems to result in a stronger general effect on white matter, that is, a greater increase in the number of synapses and a wider average axon diameter. Music practice seems to give rise to an increase in white matter specifically in those brain areas that are of relevance to the production of music [23].

We consider that these factors are also crucial parts of the artistic process: when we move to music or play an instrument, we begin to reflect and think in a more embodied way. We learn to adapt our movements and synchronize our bodies with emerging states (emotions) elicited by music, or with collaborators around us. As activities that support greater self-awareness and consideration for other perspectives, we argue that music and dance can support a mind shift towards sustainability.

Investigating this parallel further, the purposes of this review are as follows:

(1) To understand the potential for music and dance to be used to initiate and facilitate change towards inner sustainability;
(2) To review a range of research in which music and dance have been used to transform inner (internal) and outer (external) changes.

## 2. Neurobiological Research

We do not often think of the mechanisms behind the physical coupling between external and internal processes. How a strong, sudden, and unexpected experience of musical improvisation “got under the skin” has been illustrated by Vickhoff et al. [33]. In order to record physiological reactions while listening to a pianist’s improvised playing, the listener was subjected to the continuous recording of their heart rate, skin conductance (sweating) and skin temperature. The initial improvisation was calm, with a slow rhythm and harmonic chords. However, all of a sudden, the music’s character changed, with a faster rhythm and unpredictable chords. The listener experienced goosebumps during this part of the recording. Bodily changes were recorded successively. At first, their heart rate started to rise. Then, their heart rate variability decreased. Almost at the same time, their sweating started to increase. Forty seconds later, their skin temperature had decreased by 0.7 centigrade. The goosebumps were unexpected. We hardly ever know when such reactions will happen. However, this physiological experiment illustrates in a clear way how music literally “gets under the skin”. The fact that it is increasingly possible due to advances in psychophysiology to describe in concrete terms how the connections between external and internal processes arise should make it easier to convince sceptics about the importance of the musical environment for bodily functions.

While the evidence seems promising, it is important to bear in mind that a lot of it is based on correlational studies. Some schools of thought believe that it is important to focus on “precise” mechanisms to be able to learn more about changes in relation to sustainability. Studies that inform us about causality may help us to understand which parts of music and dance are important for inner change, what the necessary “dosage” is, for how long effects can still be observed and how an individual’s starting position (preconceptions about sustainability) affects the course of their transformation. It is important to emphasize that one intervention that works in one given situation or context could have an adverse effect in another.

### 2.1. Music and Dance Promote Greater Self-Awareness

There is a link between dance movements and the regulation of emotions. For example, in a study based on the Swedish Twin Registry, it was shown that subjects who have had extensive practice in dancing have an elevated ability to communicate their feelings compared to other people [34]. In the same vein, in subjects who have been practicing music extensively throughout their lives, the ability to regulate emotions is better than in others [13].

In a study initiated by a teacher of eurhythmics, Göran Krantz [35], ‘naïve’ (not systematically trained) participants were asked to listen to different diatonic chords—for example, minor second, major second, minor third, major third, reaching major seventh and a perfect octave. Participants were played these chords in a random order and instructed to move spontaneously in response to the music. At a group level, the participants’ choices of movements seemed to coalesce into a pattern. For instance, the major third (a harmonic diatonic chord) was associated with an embracing movement, while the major seventh (perceived by most naïve listeners as disharmonic) incited disorganized rapid movements [35]. In a subsequent experiment with continuous ECG recording [36], more direct comparisons were made between the major third and the major seventh: the results showed that the latter chord was more often than not randomly associated with a short bout of irregular heart rhythm. Intriguingly, this heart reaction was only observed when the participants were asked to remain sitting and not to respond through movement and was disrupted when movement was allowed. The effects of the dancing and the “music” are also likely to interact with the social context of listening, our preconceptions and expectations, as well as our previous experiences of music. However, certain types of movement responses seem to be affected by the characteristics of the sound. For example, people often respond to a clear pulsating rhythm with whole-body movements [37]. Music associated with movement has also been shown to increase motivation, trigger emotions, stimulate active presence, stimulate the constructive regulation of feelings, raise achievement, and help individuals synchronize their movements with one another [38,39]. Dance and music may have the ability to facilitate increased feelings of self-trust. Children who start dancing to music early have been shown to have a high level of trust in their own body [34].

The link between movement and music is also interesting from the embodied cognition perspective. The common coding theory [40] stipulates that the planning or execution of an action (e.g., planning to pluck a string) and the perceptual consequences of this action (feeling the string against your finger, hearing the sound it makes) are coded similarly in the brain. When we engage in these actions, both the sensory and motor brain areas are activated simultaneously. There is a constant flow of information going in the direction of action–perception, where a person tries to predict the sensory outcome of an action, and the perception–action direction, where a person tries to estimate which motor commands are necessary to produce the incoming sensory information [41].

While most studies report positive effects of music and dance on self-awareness, there are also some that warn about the negative effects. Serrano and colleagues [42] compared young musicians and ballet dancers with children who did not practice either, and found that ballet dancers scored higher in psychological inflexibility (cognitive fusion and experiential avoidance). They explained the results through the high demands of ballet training; the environment is often highly critical, and perfectionism is very common. There is also pressure to maintain a low body weight, which puts dancers at greater risk of eating disorders and body image distortion. We did not find any studies that show these risks for amateur dancers who engage in dance in their free time. We believe that dancing activities with the goal of supporting positive inner change would not be risky in this sense, because it seems that a high-pressure environment is the main contributor to poorer mental health rather than dancing itself.

### 2.2. Music and Dance Promote Learning

There are case reports describing the beneficial effects of dance and music on cognitive ability, including the capacity to learn, understand and remember [14]. Children with dyslexia can benefit markedly in their ability to read and write through teachers’ skillful use of music as an educational aid [43]. The addition of dance to music further enhances the improvement of cognitive functions as it may ‘wake up’ working memory and improve memory for codes and telephone numbers, for instance [44]. Musical activities provided by schools have been shown in practical experiments to help with class cohesion, raise grade averages and have positive emotional effects on pupils, although these findings might be difficult to generalize [45,46].

In a study based on the Swedish Twin Registry, with more than 10,000 participants, it was possible to identify music-discordant monozygotic twins in the 27–54 age range. One of the twins in a pair had had lifelong, extensive experience of piano playing, and the other did not have such an experience. The difference between siblings exceeded 1000 h of piano practice. Each twin may have started piano playing as a child, but one of them soon stopped, whilst the other continued. This provides a unique possibility to study the effects of musical instrument practice on the brain since it matches the genetic factors of the comparator subjects [47]. Using magnetic resonance imaging, the researchers identified areas in the brain that differed systematically between the ‘playing’ and the ‘non-playing’ twin. Most of the differences in the piano-discordant twins were clearer in the left hemisphere of the brain. Part of this difference, the researchers argue, may be because piano playing generally demands more advanced activity using the right hand. The results showed an increase in white matter in the brain of the playing versus the non-playing twin. In addition, in the playing twin’s brain, there was an increase in the thickness of the grey matter in areas associated with motor functions in the fingers and with regard to hearing—that is, a difference in the development of nerve cells. From this research, we can imagine that areas in the brain exposed to practice are also the areas that are strengthened [47].

Another finding of this unique twin study was that the playing twin had a more developed corpus callosum. This is the area of the brain that connects the left and the right brain hemispheres. This is consistent with the observation in other data that the Twin Registry holds that subjects who have practiced music extensively in life are more likely to have a good ability to handle emotions [13]. The corpus callosum is known to play a central role not only in coordinating movements in the two sides of the body, but also in handling emotions [48]. The coordination of the left and right sides of the brain could be an important factor in the development of pro-social behaviors. Good connection between the two halves of the brain contributes to the coordination of ideas and the enactment of those ideas. To be able to coordinate movements with emotions is also an advantage when it comes to facilitating long-term solutions that may involve difficult decision making and changes in habitual behavior. Genetic factors are also important for our embodied experience of music. They help determine the shapes and sizes of our bodies, through which we engage in dance and music, as well as the capabilities of the organs involved in the perception and processing of the stimuli involved. However, even in individuals with identic genetic material, we see that engagement with these art forms helps shape and refine how they interact with the world.

### 2.3. Music and Dance Promote Care for Others

Another important piece of the puzzle may be endorphins, which are linked to social bonding and stimulated by dancing [49] and making music together [50]. People who dance or make music together feel more connected and trust each other more.

Musical training can also encourage the development of pro-social behaviors and empathy in children [51,52] and even infants [53]. While the mechanism behind this process is still not fully known, researchers are finding structural differences in the brains of musicians and dancers that might be able to explain their enhanced empathic abilities. Gujing et al. [54] found increased functional connectivity in the insula, a part of the brain that is strongly involved in empathic processes. These patterns were connected to participants’ empathy scores and the authors suggested that music and dance training might enhance the function of the insula, resulting in superior affective sensitivity.

While such structural changes can take years to emerge, interpersonal entrainment, or the process of synchronizing one’s movements with other people, can affect pro-social behaviors practically instantaneously. People who synchronize their movements show more compassion for each other and are more likely to help and trust one another [55,56,57]. People remember the faces of people who they moved in sync with better than the faces of those who were out of sync with them [58]. Entrainment seems to be especially important when individuals engaging in it perceive themselves to be from different groups (different universities, nationalities, ethnic groups etc.). After entraining, individuals have an increased sense of joint identity and are more willing to cooperate [59] and these effects are stronger after people from different socio-cultural backgrounds move in sync [60]. This is why music and dance are important when discussing questions about sustainability. People from different sociocultural backgrounds experience the problems referred to in the 2030 Agenda very differently. Engaging in music and dance activities could therefore help different people feel more connected and prepared to help one another. Entraining to another’s movements does not only facilitate motivation to pursue common goals, but also seems to aid the ability to pursue such goals [61]. In an experiment carried out by Valdesolo and colleagues [61], moving in synchrony positively influenced perceptual and motor abilities in a perceptual sensitivity task.

Interpersonal synchrony seems to be important in social relationships from a very early age. Nine-month-old infants are more likely to reach for a teddy bear that was rocked in synchrony with them than one that was rocked asynchronously [62]. Fourteen-month-old infants are more likely to help the experimenter and provide help more quickly if they move in synchrony with them [63,64,65,66]. Similar results occur in peer relationships: four-year olds who engage in synchronous activity are more likely to be helpful and cooperative [67,68]. For more detail about the facilitation of prosocial behaviors through entrainment in children, see [69].

A striking example that shows how the teaching of drum playing can be used in order to improve the environment in a school can be found in the experiment “We beat drums, not one another” [45,46]. This experiment builds upon the simple idea that performing rhythmic music with strong movement components can increase cohesiveness within a group. It was performed in a school with pronounced problems with discipline. The pupils who took part had poor grades and only a low percentage of the oldest pupils in the school passed the requirements for high school. In this school, the decision was taken that all pupils from the youngest to the oldest should receive compulsory teaching in drum playing once a week for the duration of a year. The pupils also performed concerts for one another and for their parents and teachers. The study found that drum playing had strong emotional effects on the pupils, that disruptive behavior decreased among the pupils, less damage was inflicted upon the school’s built environment, and the participants’ grades improved. Musical activities can thus support care for one’s environment and peers and they do so even between diverse groups of people [70,71,72].

### 2.4. Music and Dance Promote Wellbeing

In a recent systematic review carried out by McCrary and colleagues [73], music and dance participation were associated with positive outcomes in 17 different health domains. They found that music and dance seem to help with social wellbeing, mental health, self-reported health and wellbeing, physical fitness and function, immune function, cognition and body composition, among other benefits. Some of the benefits are associated with physical activity, which can range (for both music and dance) from low to high intensity. Both art forms are also affordable to implement and could thus help sustainably support the health of our society.

Art-related activities, including dance and music, have also been successfully integrated into workplaces to help reduce stress levels and prevent burnout [9]. Such implementations can have important “ecological ripple of effects”, as authors call them, transferring from the employees who participated to the department or ward they belong to and into the overall work environment.

Quiroga Murcia et al. [74] showed that the plasma concentration of the stress hormone cortisol decreases during tango dancing both with and without a partner. In the same study, the authors showed that the plasma concentration of testosterone increases during tango dancing when both a partner and music are present. Testosterone belongs to an important group of agents that counteract the catabolic effects of stress and bring about anabolism or regeneration. This is the case as long as the concentration of these hormones is physiologically regulated and not artificially manipulated by the addition of external anabolic hormones (as far as we know, such manipulation is not prevalent in dancers). Testosterone also has an important role both in men and women in recovery processes, such as the repair and replacement of worn-out cells. The concentration of this hormone increases in both the saliva and blood when we feel well.

In a similar study in a musical environment, an increased concentration of testosterone in saliva was observed during the first six months in subjects who started to sing once a week in a choir. This change was not observed in a randomly selected control group that was participating in lectures and group discussions during the same period [75].

Paired dancing may have additional positive effects caused by touching. During touching, the brain may excrete the hypophyseal posterior lobe hormone oxytocin, which reduces anxiety and pain. Oxytocin also seems to have a central role in the creation of cohesiveness, and so dancing with a partner may strengthen social bonds [76].

Physical activity positively stimulates the immune system, muscle strength and physical condition. For instance, in mild to medium severe depressive disorders, regular physical exercise has been shown to be as effective as anti- depressive medication and cognitive behavioral therapy in reducing depressive symptoms [77]. In a follow-up study that utilized a random control design, the effect of access to regular free dance activities on depressive and psychosomatic symptoms was examined in teenage girls who suffered from depressive and/or psychosomatic disorders [77].

Multimodal cultural interventions, where music and dance are mixed with other cultural activities such as interactive theatre, vocal improvisation, drawing and mindful training, have been studied in relation to the health effects experienced by women experiencing burnout [78]. The results after this multimodal cultural intervention show a decrease in symptoms of exhaustion and in the alexithymia scores in the intervention group compared to the control group.

Music and dance also seem to be beneficial as activities for older adults. They seem to improve health, quality of life and life satisfaction [79,80,81]. If they do not improve quality of life, they help to maintain it [82,83]. There is also some evidence of memory, cognitive attention and cognitive flexibility improvements [84,85,86,87,88]. For a more detailed review of the wellbeing- and health-related outcomes of music and dance interventions for older adults, see [89].

## 3. Interventions Using Music and Dance

### 3.1. Promoting Self-Awareness—Emotional Recognition and Expression (Emotional Intelligence)

The city of Kiruna in Sweden has been preparing for the geographical movement of the whole city to a new location. This is because the ground in the area is filled with iron ore and deep mines have been arising under the city, increasing the threat of avalanches. The physical relocation of an entire city is a dramatic social as well as physical process, particularly for the children in the area. In preparation, the city decided to create a forum for children to dance in order to use dance activities and local musicians as a method for creating a bridge between the old and the new during this transition. The symbolic expression of dance and music has enabled children to create their own representations of the transition. Important bodily memories from the old location will also be able to transfer to the new location and create trust in the new city. During this specific intervention, all children danced together—first in the old town hall and, thereafter, together again in the new town hall. Circle dances and Saami dances were chosen to facilitate this.

In a research project investigating the relationship between art therapy and chronic pain, female patients with fibromyalgia undertook treatment involving a dance program that focused on improvised movements [14]. The findings of the study showed that participants who danced together for six months became better skilled in communicating their emotions than those who did not undertake the dance program.

A key concept of this research, “alexithymia”, is a factor comprising three different components: (1) the ability to differentiate emotions, (2) the ability to put words to different feelings, and (3) the ability to externalize through reading other people’s emotions and behaviors. According to a subsequent study [34], dance interventions were also shown to be associated with the third factor (ability to externalize).

### 3.2. Promoting Learning—Creativity

Listening to music seems to support creative thinking [90,91,92]. An additional channel through which music and dance can support a more sustainable society is through the act of improvisation. Improvisation could also support divergent thinking (which is needed for creativity) or the exploration of new possibilities and solutions and the expression of divergent opinions [93,94]. A democratic environment and novel solutions to problems are of great importance to achieving greater sustainability. During improvisation, traditional elements are being challenged and novel elements are emerging. In a world where information is boundless and changes are quick, we argue that improvisation is important because it shows “value in searching for, rather than stipulating what is right” [95] (pp. 43). We argue that it is worth further exploring whether facing complexity and uncertainty in this way could aid in the process of developing a sustainable mindset.

### 3.3. Promoting Empathy—Openness to the Unfamiliar, Recognizing Emotions of Others

Cook et al. [96] used group music-making to positively affect the attitudes and behaviors of neurotypical children towards their peers with autism. Conz and Slaughter [70] developed the project Uprooted, composed of a screen dance and community workshops, where they used dance to present the experiences of immigrants from different countries. They used it to elicit what they called “kinaesthetic empathy” in both viewers and participants of the workshops. Synchronizing one’s movements with a member from a different group seems to lower the level of prejudice against that group [97]. Similarly, listening to music from another culture lowers prejudice against that culture [71]. Cook [98] writes that people often use music to gain insight into different cultures and subcultures. However, when it comes to empathy, it is not only understanding the position (cognitive) or the feeling of emotions (affective) that is important. For change to happen, there must be a desire to promote the wellbeing of the other (motivational) [99].

### 3.4. Promoting Pro-Social Behaviors (Social Cohesion: Others and Social Environment)

Colverson et al. [100] report that students engage in more empathic decision making when exposed to music as compared to a no-music setting. Reddish et al. [101] found that not only were students who performed a synchronized movement task (as opposed to asynchronous) more likely to help a stranger from the same university (demonstrating in-group pro-sociality), but they were also more likely to help a student from a different university (out-group pro-sociality). Similar effects can be observed even in small children [68,102].

A large study in Switzerland [103] was conducted with fifty-two schools, in which pupils were allocated randomly to two groups that were given extra music classes or, alternatively, experienced the regular curriculum without music. The study evaluated the behaviors and achievements of the comparative pupil groups after a period of three years. A clear result of the research was that extra music classes contributed to better social cohesiveness among pupils compared to those who were not offered the additional classes. Following from these findings is the notion that music education or practice could contribute more broadly to the development of social cohesiveness. Social cohesiveness is potentially one important element of pro-social behavior, contributing to the neutralization and counterbalancing of competitiveness, which, from a broader societal perspective, could be an antecedent of unsustainable behaviors.

### 3.5. Promoting Wellbeing—Reduction in Stress, Reduced Likelihood of Exhaustion and Burnout

A study that could be regarded as a follow-up to the Swiss school study was performed in Stockholm [104], comprising pupils in the fifth and sixth grade. They were monitored via assessments of saliva cortisol before the start of the fall semester, then in December, and finally in May–June at the end of the spring semester. Three groups were followed, the first of which received extra music teaching for one hour every week with an emphasis on togetherness in musical exercises, the second of which received extra computer training and the third of which experienced the regular school curriculum. After the whole school year, it was evident that the music group was the only group that showed a significantly decreased saliva cortisol level during school hours at midday. Accordingly, it is possible that “designed music teaching” may have the potential to calm down a stressful school environment. This could also be important for the creation of an improved environment for learning. The dance activity also helped participants to decrease the amount of pain they experienced and increase their perception of ‘life energy’ [14].

## 4. Discussion

Encouraging practical change towards the development of a sustainable society can be very difficult, not only because of the complexity of the problems, but because many individuals might not experience these problems in their everyday lives and thus feel far removed from them. We are becoming increasingly good at gathering and presenting findings about the status of our society and our planet, but scientific evidence is frequently not enough to motivate individuals towards action [105]. As stated previously, imagination and empathy are crucial to understanding how different unsustainable practices affect other people, or how they might affect them in the future. Music and dance are two media that might be able to help us make this process easier.

Through this review, we have attempted to impart an understanding of the potential of music and dance for initiating and facilitating changes towards inner sustainability. By this, we mean an individual’s set of values, beliefs, attitudes, and emotions, which enable an individual to replenish their resources. We have attempted to provide several examples that illustrate how dance and music could facilitate mind shifts in different contexts. Music and dance may offer a way to facilitate inner change and a mind shift towards sustainable behaviors. In particular, we demonstrate how music and dance activities stimulate and engage emotions that support the development of the qualities needed for deep transformation and the emergence of inner sustainability [106]. There are still many questions regarding possible new uses of dance and music to be answered, but the evidence we have so far shows that music and dance are two modalities worth exploring in greater detail in regard to sustainability.

The 2030 Agenda presents a number of complex challenges [2]. Building on previous work, we argue that if we are unable to take care of ourselves, it is difficult to take care of others [17]. We have reviewed and synthesized a broad base of evidence to support the usefulness of the arts in promoting inner sustainability.

One thing we might bear in mind in demonstrating the power of music and dance for transformative aims is that transformation may also be sought by some toward undesirable aims. Depending on one’s political views, facilitation for good could be viewed as manipulative, or damaging. It is important to consider the ethical frames through which we make decisions about using tools and techniques that seek to shape the minds of others. Throughout history, we have seen evidence that political leaders, emperors and dictators have used music, musicians and dancers to pursue their goals and objectives contrary to what we might now consider ‘good’ intentions [107]. One way of formulating this is to say that the arts could function as amplifiers of all kinds of cognitive messages, regardless of their evil or benevolent intentions. Therefore, context will always be crucial.

From the research, the two art forms appear to have the capacity to strengthen social cohesion, nurture a sense of belonging and even lower levels of prejudice against those that are perceived as “other groups”. Creative acts, especially improvised, can promote divergent thinking and a more democratic environment, which are crucial for a sustainable society. Music and dance can contribute to—and sustain—cultural diversity as well as diversity of thought and ideas, while not dividing people. If used with benevolent goals in mind, they can create social bonds and encourage prosocial behavior between diverse groups of people [70,71,72]. The arts could therefore be regarded as something taking us further away from fixed definitions and could be an important door-opener to pro-social behaviors.

While these results seem promising, we do need to keep two things in mind. Firstly, we do not have much evidence on the robustness or duration of these effects [108] and, secondly, bonding through entrainment could also have negative consequences in certain situations. While the negative effects of music are rarely found in studies, as well as rarely systematically researched, there is some evidence of these possible negative effects. For instance, entrainment can increase in-group bias and could make it more likely for individuals to adopt the ideology of the group without critical reflection [109]. Wiltermuth [110] showed that after synchronous actions that promoted social bonding, participants were more likely to follow requests from other group members, even if that meant engaging in aggressive behavior. Lamarre and colleagues [111] showed that songs with lyrics that are negative towards specific ethnic groups can affect how participants choose to allocate funds to different ethnic groups. It is therefore important to create benevolent attitudes and frameworks that consider the whole context. One should be aware that music and dance frameworks serve as amplifiers of societal messages, irrespective of whether they are “good” or “bad”.

## 5. Conclusions

In this review, we have attempted to show how music and dance can offer a way to process and accept inner change and to facilitate a mind shift toward inner sustainable behaviors. In particular, we demonstrate how music and dance activities stimulate and engage emotions, which support the development of qualities needed for deep transformation and the emergence of inner sustainability. Music and dance can be seen as amplifiers of different messages and it is important to recognize their seemingly gentle but deep power, and to use it as another source of individual and societal resilience. While music and dance show promise in supporting inner, personal changes, we believe that structural efforts are just as important in developing a sustainable society.

It is also very important that the context surrounding the music and dance experiences are carefully designed since music and dance can also amplify counterproductive processes in the wrong circumstances.

We conclude with two examples that illustrate the significance of dance and music:

Dance and music are prohibited in some political systems. Although this is speculative, the main reason for this is probably that music and dance can empower large groups of people. This could develop into a threat to those in power.

Malaguzzi, the founder of the Reggio Emilia pedagogical system for small children, was working as a teacher in northern Italy immediately after WWII [112]. He introduced a teaching system with the goal of stimulating critical constructive thinking in children (to prevent a climate that allowed for the development of all forms of totalitarian thinking). All forms of art were central in this system—music, dance, theatre and the visual arts. We require a movement of action that provides dance and music within a constructive framework for stimulating social sustainability, and evaluation research will play a central role in this.
